# Variability in recurrent retroperitoneal liposarcomas: A case series exploring histological subtypes

**DOI:** 10.1016/j.ijscr.2025.111284

**Published:** 2025-04-12

**Authors:** Hesameddin Eghlimi, Hamidreza Movahedi, Parisa Pooyan

**Affiliations:** a Department of General Surgery, School of Medicine, Ayatollah Taleghani Hospital, Shahid Beheshti University of Medical Sciences, Tehran, Iran; b School of Medicine, Shahid Beheshti University of Medical Sciences, Tehran, Iran

**Keywords:** Retroperitoneal liposarcoma, Well-differentiated liposarcoma, Dedifferentiated liposarcoma, Recurrent liposarcoma

## Abstract

**Introduction and importance:**

Retroperitoneal liposarcomas (RPLPS) are rare soft tissue sarcomas that often present asymptomatically, leading to delayed diagnosis and challenging management. This case series highlights the impact of histological subtypes on prognosis and recurrence.

**Case presentation:**

Three male patients with recurrent RPLPS were compared. Two cases of well-differentiated liposarcoma (WDLPS) had favorable outcomes with tumor-free margins, despite recurrence in one. The third case, a dedifferentiated liposarcoma (DDLPS), presented with a larger, high-grade tumor requiring extensive resection and had a higher recurrence risk.

**Clinical discussion:**

Histological subtype, tumor size, and grade were key prognostic factors. WDLPS showed better outcomes, while DDLPS was more aggressive despite radical surgery. Recurrence remained a major concern, emphasizing the need for early detection and vigilant long-term surveillance.

**Conclusion:**

This case series underscores the variability in RPLPS presentation and outcomes, highlighting the need for individualized surgical strategies and close follow-up to improve long-term prognosis.

## Highlights

• WDLPS show better outcomes; DDLPS needs aggressive surgery and has higher recurrence risk.
• Recurrence occured despite full resection, stressing need for long-term surveillance.
• Larger, high-grade tumors like the 57×47×22 cm DDLPS case required multi-organ resection.

## 1 Introduction

Liposarcomas (LS) is the most common form of soft tissue sarcoma, accounting for approximately 15 % of all soft tissue sarcomas. [1,2] with extremities (39–41 %) and retroperitoneum (21–22 %) being common spots. [3] Retroperitoneal liposarcomas (RPLPS) arise from adipose tissue within the retroperitoneal space and are often diagnosed at advanced stages due to their insidious growth and nonspecific symptoms. [4] The incidence of (RPLPS) is estimated at 2.5 cases per million annually, with a slight male predominance and peak occurrence in the fifth to sixth decades of life. [5,6] Survival rates and recurrence vary significantly based on histological subtype and completeness of resection. [7] Well-differentiated liposarcoma (WDLPS) has a more favorable prognosis, while dedifferentiated liposarcoma (DDLPS) exhibits higher recurrence rates and worse survival outcomes. [8,9]

The clinical presentation of RPLPS is often nonspecific, characterized by abdominal distension, pain, or mass effect on adjacent organs. [10] Due to the retroperitoneum's large capacity, tumors can grow significantly before producing symptoms, delaying diagnosis. [11] Radiological imaging, including CT and MRI, plays a critical role in diagnosis and surgical planning. [12] Histopathology remains the gold standard for confirming the diagnosis and identifying the specific liposarcoma subtype.

## 2 Case presentation 1

### 2.1 Clinical presentation

A 47-year-old Iranian male presented to our department with significant abdominal enlargement that was not painful. He reported additional symptoms of post-nasal drip (PND) and urinary dribbling, with the PND occasionally causing morning vomiting.

### 2.2 Radiological and pathological findings

**Fig. 1:**
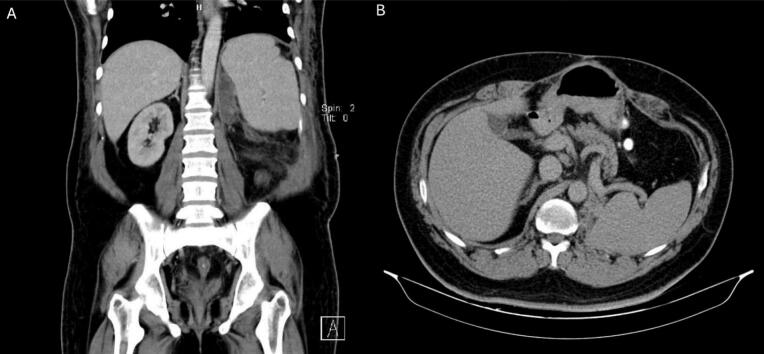
Abdominopelvic CT scan of liposarcoma case 1. A: coronal view displaying the extent of the liposarcoma in the vertical plane encasing the left kidney. B: axial view showing the transverse cross-section of the liposarcoma extending to the retroperitoneal area.

Imaging revealed a large retroperitoneal mass. (Fig. 1) Biopsy findings confirmed well-differentiated liposarcoma with a tumor size of 53 × 47 × 20 cm. Microscopic examination identified atypical lipomatous tumor components, no necrosis, and no lymphovascular or perineural invasion. (Table 1).

**Table 1:** Summary of biopsy and pathological findings.

Characteristic	Case 1	Case 2	Case 3
Specimen Description	En bloc resection of abdominal mass, kidney, and left colectomy	Wide resection of abdominal mass, small intestine, gallbladder, appendix	Wide resection of abdominal mass, kidney, intestine
Histologic Type	Well-differentiated liposarcoma	Well-differentiated liposarcoma	Dedifferentiated liposarcoma (fibrosarcoma)
Tumor Size (cm)	53 × 47 × 20	19 × 14 × 12	57 × 47 × 22
Tumor Grade	Grade 1	Grade 1	High grade
Mitosis (per 10 HPF)	0–9	Score 1	Score 3
Necrosis	No necrosis	Score 1 (<50 % necrosis)	Present
Differentiation	Atypical lipomatous tumor	Differentiation score: T	Differentiation score: 3
Margins	Tumor-free parenchyma	All margins free	All margins free
Lymphovascular Invasion	Not identified	Not identified	Not identified
Perineural Invasion	Not identified	Not identified	Not identified
Pathologic Stage	pT4 N0 Mx	Not identified	Not submitted

### 2.3 Surgical management

The patient underwent en bloc resection of the retroperitoneal mass along with nephrectomy. A left colectomy was also performed, followed by dissection of bilateral pelvic lymph nodes and colorectal anastomosis. The tumor was found to extend between the urethra and kidney, but kidney parenchyma remained free of tumor.

### 2.4 Outcomes

Postoperative recovery was uneventful, and the margins were tumor-free. Surveillance imaging was scheduled for long-term monitoring.

This case was previously reported as a standalone case report. [13] In this case series, we present it alongside additional cases to provide a comparative perspective on histological subtypes, recurrence, and treatment strategies.

## 3 Case presentation 2

### 3.1 Clinical presentation

A 66-year-old Iranian male was admitted to our department with the chief complaint of abdominal tumor recurrence and abdominal enlargement. The patient had a known history of liposarcoma and underwent surgery seven years ago, followed by recurrence one year later. He received another surgical intervention and four sessions of chemotherapy.

### 3.2 Radiological and pathological findings

**Fig. 2:**
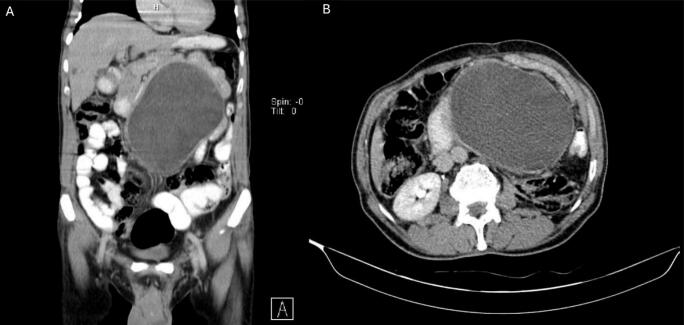
Abdominopelvic CT scan of liposarcoma case 1. A: coronal view displaying the extent of the liposarcoma in the vertical plane. B: axial view showing the transverse cross-section of the liposarcoma. A solid mass involving the entire abdomen extended to the inguinal area with is slightly more prominent on the left side. The patient previously had left nephrectomy due to the first appearance of the liposarcoma 7 years ago.

Imaging demonstrated a recurrent abdominal mass. (Fig. 2) Biopsy confirmed well-differentiated liposarcoma measuring 19 × 14 × 12 cm. Histology revealed minimal necrosis (<50 %) and no lymphovascular or perineural invasion. (Table 1).

### 3.3 Surgical management

The patient underwent wide resection of the abdominal mass, along with small intestine resection, cholecystectomy, and appendectomy. Margins were confirmed to be tumor-free.

### 3.4 Outcomes

Postoperative recovery was uneventful. Despite previous therapies, tumor recurrence remains a significant concern, and long-term follow-up is ongoing.

## 4 Case presentation 3

### 4.1 Clinical presentation

A 50-year-old Iranian male was admitted to our department with a chief complaint of a significant abdominal mass that was not accompanied by pain. The patient underwent tumor resection surgery for the first time a year ago. However, his tumor recurred a month after surgery. Four months ago, another surgery was canceled due to tumor spread, and he subsequently received 18 sessions of chemotherapy, which were ineffective.

### 4.2 Radiological and pathological findings

**Fig. 3:**
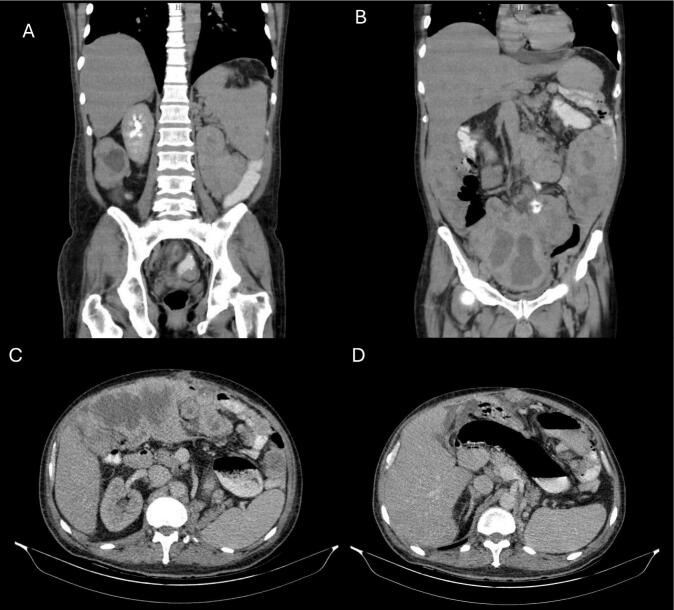
Abdominopelvic CT scan of liposarcoma case 3. A,B: coronal view displaying the extent of the liposarcoma in the vertical plane. C: axial view showing the transverse cross-section of the liposarcoma occupying abdomino-pelvic cavity was seen with the size of 350*165 from the umbilical area to the right. D: axial view showing the transverse cross-section of the liposarcoma and in incasement of left kidney. Another mass in the LUQ was seen with a previous size of 68*53.

Imaging revealed an extensive retroperitoneal mass. (Fig. 3) Biopsy confirmed dedifferentiated liposarcoma with a fibrosarcomatous component, measuring 57 × 47 × 22 cm. Necrosis was present, and the histological grade was high (Grade 3). (Table 1).

### 4.3 Surgical management

Wide resection of the retroperitoneal mass was performed, including left nephrectomy and resection of part of the intestine. Margins were confirmed to be tumor-free.

### 4.4 Outcomes

Postoperative recovery was satisfactory. The presence of dedifferentiation and necrosis warrants close monitoring for recurrence and progression.

## 5 Discussion

Retroperitoneal liposarcomas (RPLPS) are rare but represent a significant portion of soft tissue sarcomas, posing considerable clinical challenges due to their insidious growth and recurrence rates. In this case series three patients with retroperitoneal liposarcomas of varying histological subtypes, tumor sizes, and clinical outcomes, are explored emphasizing the complexities associated with diagnosis, management, and prognosis of this malignancy.

The retroperitoneum, as a common site for liposarcomas, creates significant diagnostic delays owing to the asymptomatic progression of large masses. [14] As observed in all three cases, patients typically presented with abdominal distension or mass effect without significant gastrointestinal symptoms, a finding consistent with prior reports in the literature. According to Mussi et al. [15] and Tseng et al. [16], the retroperitoneal location allows tumors to reach enormous sizes before causing noticeable symptoms, complicating timely diagnosis and increasing the risk of recurrence.

Histological subtype significantly impacts the clinical course and prognosis of liposarcoma. Well-differentiated liposarcoma (WDLPS), seen in Cases 1 and 2, is generally regarded as low-grade with a favorable prognosis; however, local recurrence remains a major issue, as observed in Case 2, where the patient experienced tumor recurrence twice despite surgical interventions and chemotherapy. Dedifferentiated liposarcoma (DDLPS), as seen in Case 3, exhibits higher histological grades, aggressive behavior, and worse outcomes. This finding aligns with the work of Crago et al., who demonstrated that DDLPS carries higher recurrence and metastatic rates compared to WDLPS. [17] Furthermore, the fibrosarcomatous component in Case 3 underscores the heterogeneity and aggressive nature of dedifferentiated tumors.

Emerging evidence suggests that not all dedifferentiated liposarcomas exhibit high-grade features. A subset of these tumors, previously classified strictly as aggressive malignancies, may demonstrate lower-grade histology, making their differentiation from well-differentiated liposarcomas more challenging. This evolving understanding has significant clinical implications, as low-grade dedifferentiated liposarcomas may have a more indolent course than previously thought. The differentiation between WDLPS and low-grade DDLPS remains a diagnostic challenge, requiring advanced imaging and histopathological assessment. [18,19]

According to findings of Henricks et al., the concept of low-grade dedifferentiation was introduced to describe fibrous areas within atypical lipomatous tumors that exhibit increased cellularity and mild atypia, but a low mitotic rate. While some cases of low-grade dedifferentiation have shown metastatic potential, the majority follow a clinical course similar to well-differentiated liposarcomas. [19] Evans further expanded on this, describing cellular atypical lipomatous tumors that contain areas of increased cellularity but lack the high mitotic rate of classic dedifferentiation. These findings underscore the need for precise histopathological classification to avoid overtreatment or undertreatment. [18]

PET scans, particularly FDG PET/CT, play a crucial role in differentiating well-differentiated from dedifferentiated liposarcomas by identifying areas of increased metabolic activity. This aids in biopsy guidance, surgical planning, and post-surgical monitoring. While PET/CT improves tumor characterization and helps detect early recurrences, its limitations include false positives from inflammation and inconsistent FDG uptake in low-grade dedifferentiation. Despite these challenges, PET scans enhance diagnostic accuracy and treatment strategies. [20]

Surgical resection remains the cornerstone of treatment for retroperitoneal liposarcomas, with complete macroscopic resection offering the best chance for disease control. [21,22] In all three cases, extensive surgeries were performed, including nephrectomy, colectomy, and vascular repairs, reflecting the invasive nature of RPLPS and the need for multi-organ resection to achieve clear margins. These findings echo results from Bonvalot et al., who highlighted the importance of achieving R0 resection for improved local control and survival outcomes. [23] Vascular involvement, as seen in Cases 1 and 3, necessitated repair and grafting, underscoring the challenges posed by tumors encasing major vessels. This aligns with findings from Tseng et al., who reported that vascular involvement often correlates with more complex resections and increased surgical morbidity. [16] Additionally, successful vascular reconstruction has been shown to contribute to prolonged survival in select cases, further emphasizing the critical role of surgical expertise in managing such cases. [24]

Despite surgical advances, recurrence remains a significant challenge in retroperitoneal liposarcomas. In our series, the patient in Case 2 experienced multiple recurrences despite previous surgeries and chemotherapy, highlighting the aggressive nature of the disease and the importance of long-term surveillance. Most recurrences occur within two years of initial resection and are often asymptomatic, necessitating vigilant follow-up with imaging, as subtle recurrences can mimic post-surgical fibrosis or normal retroperitoneal fat. [[25], [26], [27], [28]] Early detection is crucial, as complete re-resection can be achieved in up to 90 % of cases, improving outcomes, whereas late diagnoses often result in incomplete resections and further recurrences. [29,30] Current guidelines recommend imaging follow-up with MRI or CT every 3–4 months for the first two years, 4–6 months for years 3–5, and annually thereafter, as delayed recurrences are not uncommon. [31,32] This emphasizes the need for meticulous monitoring to enable timely intervention and improve survival rates.

The role of adjuvant therapies in retroperitoneal liposarcomas remains controversial. While radiation therapy has shown some benefit in improving local control for extremity sarcomas, its efficacy in RPLPS is limited due to concerns of toxicity to adjacent organs. In Case 2, chemotherapy did not prevent recurrence, highlighting the limited response of WDLPS and DDLPS to systemic therapies. As discussed by Singer et al., WDLPS is particularly chemoresistant, whereas DDLPS may show modest responses to anthracycline-based regimens. [7] Future studies are needed to explore novel therapeutic options, including targeted therapies and immunotherapies.

Tumor size and grade are critical prognostic factors in retroperitoneal liposarcomas. In this series, tumor sizes ranged from 19 to 57 cm, with the largest tumor in Case 3 being a dedifferentiated liposarcoma. As evidenced by An et al., larger tumor size, high-grade histology, and necrosis are associated with poorer survival outcomes. [33] Notably, the absence of lymphovascular and perineural invasion in all three cases is a favorable prognostic indicator, despite the aggressive behavior seen in Case 3.

## 6 Conclusion

This case series underscores the challenges in diagnosing and managing retroperitoneal liposarcomas. Future advancements in molecular profiling and targeted treatments may offer hope for better disease control and patient survival.

This study has been reported in line with the PROCESS criteria. [34]

## Informed consent statement

Informed consent was obtained from all subjects involved in the study.

## Ethical approval

Shahid Beheshti university of medical sciences did not require an ethical approval for case reports and case series and only written informed consents of the patients involved in the study is required.

## Guarantor

Parisa Pooyan.

Hesameddin Eghlimi.

## Declaration of Generative AI and AI-assisted Technologies in the Writing Process

During the preparation of this work the author(s) used chat.openai in order to check the manuscript for grammatical mistakes, improve readability and language. After using this tool/service, the author(s) reviewed and edited the content as needed and take(s) full responsibility for the content of the publication.

## Funding

This research had no external funding.

## Author contribution

Hesameddin Eghlimi: conceptualization and writing the paper.

Hamidreza Movahedi: writing the paper.

Parisa Pooyan: writing the paper.

## Declaration of competing interest

The authors declare that they have no known competing financial interests or personal relationships that could have appeared to influence the work reported in this paper.

## Data Availability

No data was used for the research described in the article.
